# Identifying and recruiting smokers for preoperative smoking cessation—a systematic review of methods reported in published studies

**DOI:** 10.1186/s13643-015-0152-x

**Published:** 2015-11-11

**Authors:** Fujian Song, Tracey J. Brown, Annie Blyth, Vivienne Maskrey, Iain McNamara, Simon Donell

**Affiliations:** 1 Norwich Medical School, Faculty of Medicine and Health Science, University of East Anglia, Norwich, Norfolk UK; 2 Orthopaedic Department, Norfolk and Norwich University Hospital, Norwich, Norfolk UK

**Keywords:** Preoperative smoking cessation, Elective surgery, Postoperative complication, Participant recruitment

## Abstract

**Background:**

Smoking cessation before surgery reduces postoperative complications, and the benefit is positively associated with the duration of being abstinent before a surgical procedure. A key issue in providing preoperative smoking cessation support is to identify people who smoke as early as possible before elective surgery. This review aims to summarise methods used to identify and recruit smokers awaiting elective surgery.

**Methods:**

We searched MEDLINE, EMBASE, CINAHL, and PsycINFO, and references of relevant reviews (up to May 2014) to identify prospective studies that evaluated preoperative smoking cessation programmes. One reviewer extracted and a second reviewer checked data from the included studies. Data extracted from included studies were presented in tables and narratively described.

**Results:**

We included 32 relevant studies, including 18 randomised controlled trials (RCTs) and 14 non-randomised studies (NRS). Smokers were recruited at preoperative clinics (*n* = 18), from surgery waiting lists (*n* = 6), or by general practitioners (*n* = 1), and the recruitment methods were not explicitly described in seven studies. Time points of preoperative recruitment of smokers was unclear in four studies, less than 4 weeks before surgery in 17 studies, and at least 4 weeks before surgery in only 11 studies. The recruitment rate tended to be lower in RCTs (median 58.2 %, range 9.1 to 90.9 %) than that in NRS (median 99.1 %, range 12.3 to 100 %) and lower in preoperative clinic-based RCTs (median 54.4 %, range 9.1 to 82.4 %) than that in waiting list-based RCTs (median 70.1 %, range 36.8 to 85.0 %). Smokers were recruited at least 4 weeks before surgery in four of the six waiting list-based studies and in only three of the 18 preoperative clinic-based studies.

**Conclusions:**

Published studies often inadequately described the methods for recruiting smokers into preoperative smoking cessation programmes. Although smoking cessation at any time is beneficial, many programmes recruited smokers at times very close to scheduled surgery so that the benefit of preoperative smoking cessation may have not been fully effected. Optimal delivery of preoperative smoking cessation remains challenging, and further research is required to develop effective preoperative cessation programmes for smokers awaiting elective operations.

**Electronic supplementary material:**

The online version of this article (doi:10.1186/s13643-015-0152-x) contains supplementary material, which is available to authorized users.

## Background

Tobacco use remains a serious global public health problem [1]. It has been suggested that surgery may offer a powerful “teachable moment” for patients to quit smoking [2, 3]. The risk of postoperative complications is much higher in smokers compared to non-smokers [4]. Smoking cessation before surgery reduces postoperative complications, and the benefit is positively associated with the duration of being abstinent before a surgical procedure [5]. Interventions for preoperative smoking cessation may include brief advice, educational booklets, pharmacotherapy, and referral to telephone quitlines and professional smoking cessation clinics [6]. According to findings from a Cochrane systematic review, intensive smoking cessation interventions provided 4–8 weeks before surgery may be more effective than less intensive and later interventions in terms of postoperative complications and long-term smoking abstinence [7].

A key issue in providing preoperative smoking cessation support is to identify people who smoke as early as possible before elective surgery. A Cochrane systematic review of methods for recruiting smokers into cessation programmes did not include any study recruiting smokers specifically for preoperative smoking cessation [8]. Cost-effective strategies for recruiting smokers awaiting elective surgery are likely to be different from strategies for identifying and recruiting smokers in the general population. The National Institute for Health and Clinical Excellence (NICE) in the United Kingdom (UK) recommended that all smokers awaiting elective surgery should be identified, and that the smoking cessation interventions could be provided “at the point of referral in primary care, during secondary care consultations and/or at preoperative assessment” [9]. Methods for identifying and recruiting smokers for preoperative smoking cessation may be defined at least according to where (e.g. primary care or preoperative evaluation clinics), when (time before surgery), and by whom (e.g. general practitioners, nurses, or surgeons). However, it is still unclear which methods are most appropriate and feasible in practice to recruit smokers into preoperative smoking cessation programmes. This systematic review aims to summarise methods that could be used to identify and recruit smokers awaiting elective surgery.

## Methods

This systematic review of methods for identifying and recruiting smokers into preoperative smoking cessation programmes was conducted according to an outline protocol (Additional file 1 and Additional file 5).

### Inclusion and exclusion criteria

We included prospective studies that (1) compared different methods of recruiting smokers for preoperative smoking cessation programmes or (2) evaluated preoperative smoking cessation programmes.

We excluded the following studies:

- Studies in which no preoperative smoking cessation interventions were provided
- Studies with a retrospective design
- Studies not published in full or published in languages other than English

### Literature search and study selection

We searched MEDLINE, EMBASE, CINAHL, and PsycINFO, and references of relevant reviews (see Additional file 1 for search strategies used) to identify relevant studies. Literature searches were conducted in May 2014, and no date limitation was applied.

Two reviewers independently screened titles and abstracts of all references identified from searching bibliographic databases. Full texts were obtained and assessed according to the inclusion and exclusion criteria by two independent reviewers. Any disagreements were resolved through discussion or the involvement of a third reviewer if required.

### Data extraction and evidence synthesis

One reviewer extracted and a second reviewer checked data extracted from the included studies. The following data were extracted from the included studies: the study design; the type of surgical procedure; country and setting (hospital or general practice); the methods for identifying and recruiting smokers (when, where, how, and by whom); the type of preoperative smoking cessation interventions provided; the outcome measures; the target quit duration before surgery; the number of recruited preoperative smokers; and the recruitment rate (defined as the proportion of recruited smokers in all eligible smokers identified). We contacted authors of some studies by emails to request additional information on missing or unclear data when available. Data and information extracted from included studies were presented in tables and narratively described.

### Ethics and consent

This study is a literature-based systematic review, without directly involving human subjects. Therefore, the approval of an ethics committee is not required and informed consent for participation is not applicable.

## Results

**Fig. 1:**
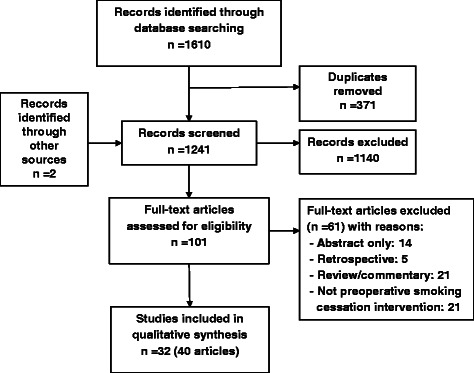
Study selection flow diagram

After screening 1612 references initially located by searching literature databases, we examined 101 full text articles and included 32 relevant studies (Fig. 1 and Additional file 4). Table 1 shows the main characteristics and methods used in the included studies to identify and recruit smokers for preoperative smoking cessation. We obtained additional information from authors of ten studies (Table 1). More details on study characteristics of the included studies, including preoperative smoking cessation interventions, outcome measures used, and additional publications of the same studies, are provided in Additional file 2. A list of full text articles excluded and reasons for exclusion are available in Additional file 3.

**Table 1:** Methods used in the included studies to identify and recruit smokers for preoperative smoking cessation

Study: author year (country)	Surgery: no. of smokers (recruitment rate)	Recruiting method	How (and when) recruited before surgery	Target quit period before surgery
Randomised controlled trials (RCTs)				
Andrews et al. 2006 [10]^a^ (UK)	Elective surgery 102 (NA)	At the routine 4 weeks preoperative appointment, a nurse asked all patientswho smoked if they would be interested in the trial. If patients were interested, a researcher asked patients to sign a consent form.	Preoperative clinic (about 4 weeks)	1–2 weeks
Glasgow et al. 2008 [11] (USA)	Multiple procedures 391 (36.8 %)	Smokers about to undergo surgery or diagnostic procedure were identified usingelectronic medical records system and received a personalised introductory letter about the study from the chief of preventive medicine (with an “opt-out” postcard). 1–2 weeks prior to procedure date, trained telephone interviewers contacted smoking patients who did not decline via postcard.	Screening of electronic medical records (1–2 weeks)	Smoking reduction 1–2 weeks before surgery
Lee et al. 2013 [12] (Canada)	Elective surgery 168 (43.0 %)	Patients scheduled for elective surgery having a pre-admission clinic appointment at least 3 weeks before surgery were screened using a questionnaire for smoking status. The researchers’ interaction included informed consent. Nurses in the pre-admission clinic randomised participants to groups on the day of enrolment.	Pre-admission clinic (anaesthesia) (≥3 weeks: median 24 days, range 22 to 31)	≥1 week
Lindstrom et al. 2008 [13] (Sweden)	General or orthopaedic 117 (58.2 %)	Participants were enrolled by study nurses or by the treating surgeons >4 weeks prior to surgery. 586 were planned to be recruited, but the trial was stopped early due to a low rate of recruitment and a high refusal rate (with only 117 smokers recruited).	Not explicitly reported (≥4 weeks)	4 weeks
McHugh et al. 2001 [14] (UK)	Coronary artery bypass surgery 121 (85.0 %)	Consecutive patients were identified within 1 month as they were added to the waiting list. Patient’s GPs were contacted by letter for consent of their patients to be recruited to the study. Unclear about who identified and recruited smokers. The mean (SD) waiting time was about 8.4 (2.7) months.	At the time of waiting list placement (mean waiting time 8.4 months, SD 2.7)	Unclear
Moller et al. 2002 [15]^a^ (Denmark)	Hip and knee replacement 120 (72.3 %)	Daily smokers scheduled for primary elective hip or knee alloplasty were recruited 6–8 weeks before scheduled surgery. A project nurse explained the study detail to patients.	Preoperative clinic (6–8 weeks)	6–8 weeks^a^
Myles et al. 2004 [16]^a^ (Australia)	Elective surgery 47 (10.7 %^a^)	Research staff screened the elective surgery waiting list to identify smokers expected to undergo surgery within an 8–14-week timeframe and asked potentially eligible participants to contact research staff for further details. (The original protocol was to investigate smoking cessation at the time of hospital admission for surgery, but the study protocol was revised due to a low rate of recruitment and a high dropout rate.)	Screening of waiting list (8–14 weeks: median 120 days, IQR 60 to 120)	About 28 days^a^
Ostroff et al. 2013 [17] (USA)	Cancer surgery 185 (70.1 %)	Participants with newly diagnosed cancer scheduled for surgery (>7 days from study entry) were screened via the electronic medical record and recruited from surgical clinics, by a trained research assistant.	Screening of electronic medical records (≥1 week)	>1 day before inpatient admission
Ratner et al. 2004 [18] (Canada)	Elective surgery 237 (56.7 %)	Patients admitted for pre-surgical assessment (1–3 weeks before surgery) were screened for eligibility by registered nurses.	Preoperative clinic (1–3 weeks)	>1 day before surgery
Shi et al. 2013 [19]^a^ (USA)	Elective surgery 169 (92.3 %^a^)	As part of routine preoperative evaluation (POE), patients provide information on smoking behaviour. The median time from study assessment at POE to surgery was 1 day (IQR 1 to 3). Smokers were identified by clinical POE personnel on a convenience basis. Consent by study personnel was obtained after study procedures were completed.	Preoperative clinic (anaesthesia) (median 1 day; IQR 1 to 3)	<1 day (on the day of surgery)
Sorensen et al. 2007 [20] (Denmark)	Herniotomy 180 (90.9 %)	Smokers scheduled for elective open incisional or inguinal day-case herniotomy were included (about 3 months before surgery). Recruiting method was not described, although the involvement of a study nurse was mentioned.	Not explicitly reported (about 3 months)	>4 weeks
Sorensen and Jorgensen 2003 [21] (Denmark)	Open colorectal 60 (74.1 %)	At the time of diagnosis and selection of operative procedure, patients who smoked daily and were scheduled for an open colonic or rectal surgery were included. The study was originally planned as a 5-centre trial of 300 smokers. Four centres failed to enrol participants after initiation, and recruitment was continued at the remaining centre until an equal number of participants in both groups were enrolled (*n* = 60). Unclear about how smokers were identified and recruited.	Not explicitly reported (>2–3 weeks)	2–3 weeks
Thomsen et al. 2010 [22] (Denmark)	Breast cancer 130 (50.2 %)	Women scheduled for breast surgery were included (3–7 days before surgery). Methods for identifying and recruiting smokers were unclear.	Not explicitly reported (3–7 days)	>2 days
Warner et al. 2011 [23]^a^ (USA)	Elective surgery 300 (68.5 %)	Current smokers were recruited from the preoperative evaluation clinic in preparation for surgery on a convenience basis. Recruitment occurred when appropriate research and clinical personnel were available. Time from preoperative evaluation to surgery: median 1 day, IQR 1–4 days.	Preoperative clinic (anaesthesia) (median 1 day; IQR 1 to 4 days)	About 1 day
Warner et al. 2005 [24] (USA)	Elective surgery 121 (9.1 %)	Smokers were recruited from patients evaluated at the preoperative evaluation in preparation for surgery.	Preoperative clinic (anaesthesia) (<1 week)	About 1 day
Warner and Kadimpati 2012 [25]^a^ (USA)	Elective surgery 46 (52 %^a^)	Current smokers were recruited from the preoperative evaluation clinic in preparation for surgery on a convenience basis.	Preoperative clinic (anaesthesia) (<1 week)	About 1 day
Wolfenden et al. 2005 [26] (Australia)	Non-cardiac elective surgery 210 (82.4 %)	Patients at high risk were booked to attend a non-cardiac preoperative clinic 1–2 weeks before their scheduled procedure. Participants completed a computerised assessment, and those identifying themselves as smokers were recruited by a research assistant.	Preoperative clinic (1–2 weeks)	>1 day before admission
Wong et al. 2012 [27]^a^ (Canada)	Elective non-cardiac surgery 286 (29.6 %)	All adult patients at the preoperative clinics scheduled for elective non-cardiac surgery were screened. Smoking patients scheduled for surgery (8–30 days before the scheduled surgery), who met the eligibility criteria were recruited.	Preoperative clinic (1–4 weeks)	>1 day
Non-randomised studies (NRS)				
Backer et al. 2007 [28] (Denmark)	Acute orthopaedic surgery 121 (60.5 %)	On the day of admission, patients admitted to acute orthopaedic wards on weekdays were routinely asked about smoking habits as part of their medical history, and a trained nurse provided a motivational counselling. A specially trained nurse in the morning briefing reviewed medical files to ensure that smokers could be contacted.	On the day of admission (about 1 day)	<1 day
Browning et al. 2000 [29]^a^ (USA)	Lung cancer surgery 25 (100 %)	Researchers recruited potential participants from a lung cancer surgery clinic’s new patient schedule, during the first clinical consultation. The participant set a quit date for 14 days later.	Preoperative clinic (first clinical consultation) (NA)	Unclear
Haddock and Burrows 1997 [30] (UK)	General or gynaecology day surgery 60 (100 %)	A research nurse implemented smoking cessation programme in surgical pre-admission clinics (7–14 days before surgery). Smokers who were willing to participate were eligible for the study.	Preoperative clinic (1–2 weeks)	Unclear
Haile et al. 2002 [31] (Australia)	Non-cardiac surgery 56 (100 %)	Prior to booking in for surgery, all patients were given a surgical risk assessment by their GP, surgeon, or pre-admission staff. Patients at high risk were required to attend the pre-admission clinic 2 weeks prior to surgery, and a research assistant determined eligibility.	Preoperative clinic (2 weeks)	<2 weeks
Kozower et al. 2010 [32]^a^ (USA)	Thoracic surgery 23 (66.7 %)	Recruiting method was not reported. Included preoperative (23), postoperative (11), and follow-up smokers (6). A clinical research coordinator was present to facilitate the study.	Preoperative clinic (typically 3 weeks^a^)	2 weeks^a^
Kunzel et al. 2012 [33] (USA)	Urologic 38 (NA)	Preoperatively (the mean interval to day of surgery was 25 days, median 11, range 1–131 days). Recruiting method was not reported, although urologists and research staff performed the intervention.	Preoperative clinic (median 11 days; range 1 to 131 days)	Unclear
Moore et al. 2005 [34] (USA)	Urogynaecology surgery 233 (NA)	On their initial history and physical examinations, patients who admitted to smoking were recruited (>1 month prior to surgery). Unclear about how smokers were identified.	Not explicitly reported (≥4 weeks)	4 weeks
Munday et al. 1993 [35] (UK)	Elective surgery 233 (NA)	At the time of outpatient consultation (>6 weeks prior to surgery), smokers were identified and the hospital notes were marked to enable the smokers to be identified when admitted to hospital. Control smokers were participants admitted from the waiting list for elective surgery who smoked but had not been given specific advice to stop. Unclear about who identified and recruited smokers.	Preoperative clinic (>6 weeks)	≥6 weeks
Sachs et al. 2012 [36]^a^ (Canada)	Elective surgery (excluding CVD and neurosurgery and plastic) 714 (20 %^a^)	At pre-admission clinic, registration clerks identified patients who were current smokers and informed them about the programme. Eligible participants were asked by research staff if they would be willing to participate in the evaluation study.	Preoperative clinic (NA)	Unclear
Shah et al. 1984 [37] (UK)	Elective surgery 200 (NA)	In the intervention group a letter was sent with the admission note to all patients; non-smokers were asked to disregard the letter. In the control group, recruitment occurred on the day of the operation after recovery. Unclear about how smokers were identified and recruited.	Not explicitly reported (unclear)	5 days
Tonnesen et al. 2010 [38]^a^ (Denmark)	Elective surgery 57 (12.3 %)	GP (199) were invited to identify daily smoking when referring them to surgery and to refer high-risk patients to a preoperative risk reduction programme. However, only 2 patients were referred a few months after starting the programme, and additional efforts slightly increased referral to the programme (7/72). High-risk patients for elective surgery were also identified at the department of orthopaedic surgery and surgical gastroenterology (at their first contact to hospital^a^).	At the time of GP referral (about 6 weeks^a^)	About 6 weeks^a^
Walker et al. 2009 [39] (UK)	Forefoot surgery 25 (100 %)	Senior author (based at the Orthopaedic Department) reviewed all patients prior to planned surgery (approximately 6 months prior to planned surgery).	Screening of medical records (about 6 months)	Unclear
Webb et al. 2014 [40] (Australia)	Non-obstetric elective surgery 347 (99.1 %)	Printed quit-pack was sent to all adult patients (including smokers) at the time of waiting list placement for non-obstetric elective surgery. Help from waiting list staff was acknowledged.	At the time of waiting list placement (>4 weeks)	≥4 weeks
Wheatley et al. 1977 [41] (Australia)	Inguinal hernia repair 15 (NA)	Patients (15 smokers and 15 non-smokers) were assigned arbitrarily to one of three groups. Recruiting methods not reported.	Not explicitly reported (unclear)	5 days

Recruitment rate was calculated using the number of smokers recruited as numerator and the number of invited eligible smokers as denominator. Target quit period before surgery was based on recommended quit date, not necessarily reflect the actual quit period preoperatively

*NA* not available, *SD* standard deviation, *POE* preoperative evaluation, *GP* general practitioner, *CVD* cardiovascular diseases, *IQR* interquartile range, *USA* United States of America

^a^Authors of the studies responded to request for additional information. We were able to contact authors of 14 studies by emails and received response from authors of ten studies with some additional information on missing or unclear data

The included 32 studies were those that prospectively evaluated preoperative smoking cessation interventions (Table 1). No studies that compared different methods for identifying and recruiting smokers for preoperative smoking cessation were identified. There were 18 randomised controlled trials (RCTs) [10–27] and 14 non-randomised studies (NRS) [28–41]. The included studies were conducted in the USA (*n* = 10), Australia (*n* = 5), UK (*n* = 6), Denmark (*n* = 6), Canada (*n* = 4), and Sweden (*n* = 1). The majority of studies recruited patients who underwent general or mixed elective surgery (*n* = 19); other studies specified the type of surgery as orthopaedic (*n* = 3), lung or thoracic (*n* = 2), urologic or gynaecologic (*n* = 2), cancer (*n* = 2), hernia (*n* = 2), colorectal (*n* = 1), and coronary artery bypass (*n* = 1).

The methods and timing of identifying and recruiting smokers before surgery are presented in Table 1. In general, recruitment information from the included studies was limited. Studies were generally focused on the effectiveness of the interventions on smoking abstinence or on reducing postoperative complications rather than on reporting specific information on methods for identifying and recruiting smokers.

Most studies (*n* = 18) identified and enrolled smokers at preoperative evaluation or pre-admission clinics, including anaesthetic [12, 19, 23–25, 36] and surgical clinics [10, 15, 18, 26–33, 35]. Eligible smokers were usually identified and recruited by dedicated research personnel. Smokers were identified from elective surgery waiting lists (or medical records) in six studies [11, 14, 16, 17, 39, 40]. There was only one study in which the identification of smokers for preoperative smoking cessation was started at the point of referral in primary care [38]. The methods for identification and recruitment were not explicitly described for the remaining seven studies. The number of smokers for preoperative smoking cessation ranged from 46 to 391 (median 149) in the included RCTs and ranged from 15 to 714 (median 59) in the included non-randomised studies. There were a total of 5137 patients included in the 32 studies (2990 in RCTs and 2147 in non-randomised studies).

**Fig. 2:**
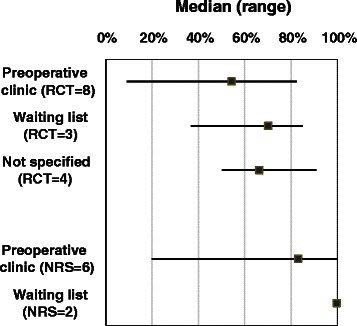
Recruitment rate by recruitment approaches. Recruitment rates are separately shown for randomised controlled trials (*RCTs*) and non-randomised studies (*NRS*). The *square point* is the median of the reported recruitment rates, and the *line across the square point* indicates the range of the reported recruitment rates

According to 24 studies that provided sufficient data, the rate of recruitment of eligible smokers ranged from 9.1 to 90.9 % (median 58.2 %) in 15 RCTs and ranged from 12.3 to 100 % (median 99.1 %) in nine non-randomised studies (Table 1). Therefore, RCTs tended to report relatively lower recruitment rate, compared with non-randomised studies. In addition, preoperative clinic-based approach tended to be associated with a lower recruitment rate than waiting list-based approach (Fig. 2). The median recruitment rate was 54.4 % (range 9.1 to 82.4 %) in eight preoperative clinic-based RCTs and 70.1 % (range 36.8 to 85.0 %) in three waiting list-based RCTs. Because of wide ranges of the reported recruitment rates and great heterogeneity in study characteristics (such as diverse patients and interventions), the observed recruitment rates should be interpreted with great caution. For example, a recruitment rate of 9.1 % was reported in a study that recruited smokers at preoperative evaluation clinics [24], whereas a much higher recruitment rate of 68.5 % was reported by the same author in a more recent study that used the same recruitment approach [23].

Smokers were enrolled into smoking cessation programmes at various time points before surgery (Table 1). The duration of preoperative recruitment of smokers was unclear in four studies, less than 4 weeks before surgery in 17 studies, and at least 4 weeks before surgery in 11 studies [10, 13–16, 20, 34, 35, 38–40]. Smokers were recruited at least 4 weeks before surgery in four of the six waiting list-based studies (67 %) and in only three of the 18 preoperative clinic-based studies (17 %) (Table 1). In one study of patients awaiting coronary surgery, smokers were identified and recruited from elective surgery waiting lists many months (mean 8 months, standard deviation (SD) 2.7) before surgery [14]. In a recent study [40], smoking cessation materials (leaflets and quitline referral forms) were posted to all patients (smokers and non-smokers) at the time of waiting list placement (>4 weeks before surgery). Investigators of a study of elective forefoot surgery recruited participants approximately 6 months before surgery [39]. In another study that identified smokers from elective surgery waiting lists, smokers were enrolled into the smoking cessation programme 8 to 14 weeks before surgery [16].

The target quit dates before surgery generally corresponded to the time when smokers were recruited (Table 1). It was unclear in six studies. In four studies conducted by the same research team, smokers were identified at preoperative evaluation clinics a few days before surgery and asked to be abstinent from smoking on the day of surgery [19, 23–25]. The target quit date was at least 4 weeks before surgery in nine studies [13, 15, 16, 20, 34, 35, 38–40].

Several of the included studies explicitly reported difficulties in identifying and recruiting smokers into preoperative smoking cessation programmes. Myles and colleagues identified smokers at the time of hospital admission for surgery but reported a low rate of recruitment and a high dropout rate [16]. Authors of two other studies reported failure to achieve the recruitment target, but the methods for recruiting smokers were not explicitly described [13, 21]. Only one study evaluated the involvement of general practitioners (GPs) in recruiting smokers into preoperative programmes, in which GPs referred only seven of the 72 high-risk patients for a preoperative programme in a period of 9 months, after some intensive efforts to encourage such GP referrals [38].

## Discussion

In this systematic review, we found that information on the recruitment of smokers was often inadequately reported in studies that evaluated preoperative smoking cessation programmes. Although smoking cessation at any time is beneficial to patients, the recruitment of smokers was often close (less than 4 weeks) to scheduled surgery and the benefit of preoperative smoking cessation for preventing postoperative complications may not have been fully effected.

Surgical treatment may be a “teachable moment” for patients to quit smoking [2]. Intensive behavioural support and pharmacological interventions are known to be effective strategies for smoking cessation [6]. However, many smokers, as well as surgeons, may not be fully aware of the risks of active smokers and the benefits of smoking cessation before surgery [3, 42]. One of the key clinical issues in providing an effective smoking cessation programme for patients having elective surgery is to identify all patients who smoke and ensure that appropriate referral pathways for cessation support are in place [9]. This review has shown that published studies of preoperative smoking cessation programmes provide inadequate information on the methods used to identify and recruit smokers. Without this information, it is impossible for other researchers to replicate and verify the effectiveness of a preoperative smoking cessation programme.

A wide range of different strategies have been used to recruit smokers into general (not preoperative specifically) cessation programmes, including mass media, telephone- or internet-based approaches, and face-to-face recruitment by health practitioners or others [8]. The recruitment of patients into preoperative smoking cessation programmes may require alternative strategies, although evidence of the effectiveness of different strategies is lacking in this area. The methods reported in the included studies were mainly (1) recruitment of smokers from preoperative outpatient clinics or (2) recruitment of smokers from waiting lists or medical notes (Table 1). However, difficulties in identifying and recruiting smokers into preoperative smoking cessation programmes have been explicitly reported in several of the included studies.

Available evidence indicated that waiting list-based approaches may be associated with a higher recruitment rate and earlier identification of smokers before surgery, compared with preoperative clinic-based approaches. However, this finding is tentative due to great heterogeneity across studies with similar approaches, and waiting list-based recruitment of smokers may not be feasible in some settings. Further research and empirical evidence are required to compare the different strategies to recruit smokers into preoperative smoking cessation programmes.

Many studies employed research personnel to identify and recruit smokers, to obtain consent, and collect data from study participants. Without the involvement of dedicated research staff, it may be uncertain whether preoperative smoking cessation interventions evaluated in studies could be implemented in normal practice. We found that the recruitment rates of eligible smokers for preoperative smoking cessation tended to be lower in the included RCTs than that in the non-randomised studies. It is assumed that preoperative smoking cessation interventions may be more acceptable to patients if such interventions are standard of care, so that the recruitment rate would be higher in clinical practice than that in research studies. However, this assumption needs to be confirmed with empirical evidence. In addition, further qualitative research may be required to understand reasons for difficulties in recruiting smokers into preoperative smoking cessation programmes, as reported in some included studies.

### Limitations

Data on smoking cessation interventions used and outcome measures in the included studies were extracted (see Additional file 2), but these data were not further considered in this systematic review. To highlight lacking of relevant research and inadequate reporting in published studies, we focused on methods for recruiting smokers into preoperative smoking cessation programmes. Because we were not aware of a suitable quality assessment tool, quality of the included studies was not systematically assessed. Identification and recruitment of smokers awaiting elective surgery may be considered as an essential component in any preoperative smoking cessation interventions. However, reporting of methods for recruiting eligible patients has not been explicitly included in the TIDieR (template for interventions description and replication) checklist for better reporting of interventions [43]. Findings of this systematic review indicate that existing checklists for reporting of interventions need to be improved.

We did not identify any studies that directly compared different methods for recruiting smokers into preoperative smoking cessation programmes. The differences in recruitment rates and target quit dates before surgery between waiting list-based and preoperative clinic-based recruitment methods were emerged from indirect comparisons across different studies, which should be interpreted with great caution. In addition, the number of relevant studies was small, and there was considerable heterogeneity in the reported recruitment rates and study characteristics. Due to resource and time restrictions, we excluded studies that were not fully published and studies that were published in languages other than English. However, methods for recruiting preoperative smokers may be more likely inadequately reported in studies that were not fully published.

## Conclusions

Published studies often inadequately described the methods for recruiting smokers into preoperative smoking cessation programmes and utilised study designs that may not be applicable to normal clinical practice. Although smoking cessation at any time is beneficial to patients, many programmes recruited smokers at times very close to scheduled procedure so the benefit of preoperative smoking cessation may not have been fully effected. Consequently, optimal delivery of preoperative smoking cessation remains challenging, and further research is urgently required to develop effective preoperative cessation programmes for smokers awaiting elective operations.

## Additional files

Additional file 1: **Preoperative identification of smokers for smoking cessation—a systematic literature review—protocol.**(DOCX 20.9 kb) Additional file 2: **The main characteristics of the included studies.** (DOCX 86.4 kb) Additional file 3: **List of excluded studies after full text examination.**(DOCX 21.5 kb) Additional file 4: **Study selection flow diagram.** (DOCX 27.8 kb) Additional file 5: **PRISMA 2009 Checklist.** (DOC 63.5 kb)

## Abbreviations

GP: general practitioner
IQR: interquartile range
NRS: non-randomised study
POE: preoperative evaluation
RCT: randomised controlled trial
SD: standard deviation
UK: United Kingdom
