# Supplementary material for: Identifying and recruiting smokers for preoperative smoking cessation—a systematic review of methods reported in published studies
Source: Syst Rev. 2015 Nov 11;4:157. doi: 10.1186/s13643-015-0152-x (PMC4642619; doi:10.1186/s13643-015-0152-x)
Supplement: Additional file 1: — Preoperative identification of smokers for smoking cessation—a systematic literature review—protocol.(DOCX 20.9 kb) [file 13643_2015_152_MOESM1_ESM.docx]

## Preoperative identification of smokers for smoking cessation – a systematic literature review - protocol

## Objectives

To summarise methods available for identifying and recruiting smokers into preoperative smoking cessation programmes.

## Inclusion and exclusion criteria

We will identify and include studies that provided evidence to answer the following two questions:

- What methods have been recommended and/or used to identify and recruit smokers for preoperative smoking cessation?
- What are effective strategies that can be implemented in practice?

Therefore, we will include studies that are prospectively designed to enrol smokers for pre-operative smoking cessation, and

- compared different methods of recruiting smokers for preoperative smoking cessation programmes (though unlikely to identify any such study), or
- reported any method of recruiting smokers for preoperative smoking cessation programmes (e.g., methods used in studies of preoperative smoking cessation interventions)
- fully published in English language

Exclusion criteria:

- Exclude studies of recruiting surgical patients for *only postoperative* smoking cessation
- Exclude *retrospective* studies
- Exclude observational studies in which *no preoperative smoking cessation interventions were provided*.
- Exclude studies not published in full, or published in languages other than English (?)

## Literature search strategy

We will search Medline, Embase, CINAHL, and PsycINFO, and references of relevant reviews, using the following strategies.

### MEDLINE STRATEGY (via OVIDSP)

1. smoking cessation.mp.

2. exp Smoking Cessation/

3. "Tobacco-Use-Cessation"/

4. "Tobacco-Use-Disorder"/

5. exp Smoking/pc,th

6. 1 or 2 or 3 or 4 or 5

7. (surgery or operation or operativ: or an?esthesia).mp.

8. exp Postoperative complication/

9. exp Preoperative care/

10. exp Patient education/

11. 8 and (9 or 10)

12. 7 or 11

13. 6 and 12

### EMBASE STRATEGY (via OVIDSP)

1. smoking cessation.mp.

2. ((smok* or tobacco or cigar*) adj3 (stop* or quit* or giv* or refrain* or reduc*)).ti,ab.

3. 1 or 2

4. (presurg* or pre-surg* or preoperati* or pre-operati* or perisurg* or peri-surg* or perioperati* or peri-operati* or prean?ethsia or pre-an?esthesia or preadmission* or pre-admission* or ((before or prior) adj2 (surgery or operation))).ti,ab.

5. 3 and 4

### CINAHL and PsycINFO (via EBSCO)

1. (smoking or tobacco or cigarette*) N3 (cessation or quit* or stop*)

2. preoperati* OR pre-operati* OR perioperati* OR peri-operati* OR presurg* OR pre-surg* perisurg* OR peri-surg* OR preadmission* OR pre-admission* OR an?esthesia OR ((before OR prior) N2 (surgery OR operation) )

3. S1 AND S2

## Study selection

Two reviewers will independently screen titles and abstracts of all references identified from searching bibliographic databases. Then full text reports of possibly relevant studies will be obtained and assessed according to the inclusion and exclusion criteria by two independent reviewers. Any disagreements will be resolved through discussion.

## Data Extraction

We will extract the following data from the included studies:

- Study ID (1^st^ author, year)
- Study design
- Type of surgical operations
- Country, and setting
- Methods for identifying and recruiting smokers
- Staff who identified and recruited smokers
- When were smokers enrolled (e.g., how many days/weeks/months before surgery)?
- Type of preoperative smoking cessation programmes/interventions
- Who delivered the pre-operative smoking cessation intervention?
- Recommended pre-operative quit date (before surgery)
- Definition of pre-operative smoking abstinence (and verified or not)
- Number of recruited pre-operative smokers

## Quality assessment

If no comparative study is included, it may not be necessary to conduct any formal assessment of study quality.

## Evidence synthesis

Data and information extracted from the included studies will be presented in tables, and narratively described.
