# Supplementary material for: Identifying and recruiting smokers for preoperative smoking cessation—a systematic review of methods reported in published studies
Source: Syst Rev. 2015 Nov 11;4:157. doi: 10.1186/s13643-015-0152-x (PMC4642619; doi:10.1186/s13643-015-0152-x)
Supplement: Additional file 2: — The main characteristics of the included studies. (DOCX 86.4 kb) [file 13643_2015_152_MOESM2_ESM.docx]

## The main characteristics of the included studies

| **Study, design and country** | **Surgery & no. of smokers** | **Recruiting method** | **Preoperative smoking cessation interventions** | **Main outcomes** |
| --- | --- | --- | --- | --- |
| **Randomised controlled trials** | | | | |
| Andrews 2006 [^1^](#_ENREF_1)  RCT  (UK) | Elective surgery.  n=102 | A nurse asked all patients who smoked if they would be interested in the trial at the routine 4 week preoperative appointment. If patients were interested a researcher asked patients to sign a consent form. | Smokers received a letter from a consultant stating "stopping smoking 1 or 2 weeks before surgery has huge benefits", in addition to a booklet and nurse advice received at routine 4 week preoperative appointment. Patients were also given contact details of the Sandwell Stop Smoking Service. Control patients received the standard booklet (giving brief advice on stopping smoking long-term but not stating temporary cessation would aid post-operative recovery) and nurse advice only. | Self-reported abstinence from smoking, on the day of operation |
| Glasgow et al 2008 [^2^](#_ENREF_2)^,^ [^3^](#_ENREF_3)  RCT  (USA) | Outpatient surgery, gastrointestinal procedure, or diagnostic procedure.  n=391 | Smokers about to undergo surgery or diagnostic procedure were identified using electronic medical records system, and received a personalised introductory letter about the study from chief of preventive medicine (with an "opt-out" postcard). 1-2 weeks prior to procedure date, trained telephone interviewers contacted smoking patients who did not decline via postcard. | The intervention was focused on smoking reduction, delivered by 4 trained interviewers without previous experience of smoking counselling. Smoking reduction interventions started 1-2 weeks before surgery but mostly postoperatively. The intervention consisted of 4 telephone counselling sessions, 4 tailored newsletters and one targeted newsletter; intervention contact was faded over time. The usual care group received three quarterly generic health education mailings. | Self-reported number of cigarettes smoked and carbon monoxide levels at 12 months. |
| Lee 2013 [^4^](#_ENREF_4)  RCT  (Canada) | Any elective surgery: including general surgery, gynaecologic, urologic, opthalmologic, otolaryngologic, and orthopedic.  n=168 | Patients scheduled for elective surgery having a preadmission clinic appointment at least 3 weeks before surgery were screened using a questionnaire for smoking status. The researchers' interaction included informed consent. Nurses in the pre-admission clinic randomised participants to groups on the day of enrolment. | The intervention included: a brief (< 5 minute) counselling session by trained nurse, "Stop Smoking for Safer Surgery" brochure; referral to a free smoking helpline (who initiated contact and aimed to have at least 4 contacts with each participant); and a 6 week supply of free transdermal nicotine replacement and medication information. Control participants received standard care (i.e. inconsistent advice). | Primary outcome: self-reported abstinence for at least 7 days before surgery combined with a CO validation on the day of surgery. |
| Lindstrom 2008 [^5-7^](#_ENREF_5)  RCT  (Sweden) | General (hernia, cholecystectomy) and orthopaedic surgery (hip or knee prosthesis).  n=117 | Participants were enrolled by study nurses or by the treating surgeons >4 weeks prior to surgery. 586 were planned to be recruited, but the trial was stopped early due to low rate of recruitment and high refusal rate (with only 117 smokers recruited). | The intervention included weekly meetings or telephone counselling with a nurse (trained in smoking cessation therapy), a telephone number to a smoking quitline, free nicotine replacement therapy. The control group received standard care. | Primary: postoperative complications. Self-reported smoking status at 2-3 weeks after surgery and at 12 months. CO measurement 2-3 weeks post-operatively. |
| McHugh 2001 [^8^](#_ENREF_8)  RCT  (UK) | Coronary artery bypass surgery.  n=121 | Consecutive patients were identified within 1 months as they were added to the waiting list for CABG. Patient's GPs were contacted by letter for consent for their patients to be recruited to the study. Unclear about who identified and recruited smokers. The mean (SD) waiting time was about 8.4 (2.7) months. | Shared care health programme targeting on multiple CHD risk factors; delivered by community based cardiac liaison nurse or general practice nurse in patient home and at the practice clinic: including monthly health education and motivational interviews, tailored to individual need. Interventions addressed smoking, physical inactivity, poor diet and excess alcohol. | Smoking status, obesity, physical activity, anxiety and depression, general health status, blood pressure, plasma cholesterol and alcohol intake at admission to hospital for surgery. |
| Moller 2002 [^9-11^](#_ENREF_9)  RCT  (Denmark) | Hip and knee replacement.  n=120 | Daily smokers scheduled for primary elective hip or knee alloplasty were recruited, 6-8 weeks before scheduled surgery. A project nurse explained the study detail to patients. | A project nurse (trained in smoking cessation techniques) provided weekly counselling and personalised NRT therapy; either smoking cessation or at least 50% smoking reduction. The intervention period was 6-8 weeks before and 10 days after operation. The control group received standard care i.e. little or no information about the risk of smoking or cessation counselling. | Postoperative complications, defined as death or those requiring treatment within 4 weeks after surgery. |
| Myles 2004 [^12^](#_ENREF_12)  RCT  (Australia) | Elective surgery (general; orthopaedic; urological; ENT; faciomaxillary; plastics; neurosurgery; other).  n=47 | Patients expected to undergo surgery within an 8-14 week timeframe were recruited from the elective surgery waiting lists. Potentially eligible participants were asked to contact research staff for further details. Screening examination and consent were undertaken by research staff. Reported that the study protocol was revised due to a low rate of recruitment and a high dropout rate. | 7 week course of bupropion versus placebo; study drug was stored and administered from the hospital pharmacy department. All patients were informed of smoking health risks particularly in relation to surgery. They were advised to give up smoking and given a "Smoking and Anaesthesia" booklet. They were telephoned 2-4 days after their quit day to encourage abstinence. | Primary: daily cigarette consumption at the time of hospital admission. Secondary: validated abstinence up until 6 months, self-reported daily cigarette consumption at 3 and 6 weeks and expired CO at hospital admission. |
| Ostroff 2013 [^13^](#_ENREF_13)  RCT  (USA) | Cancer (thoracic, head and neck, breast, GYN, urology, other).  n=185 | Participants with newly diagnosed cancer scheduled for surgery (>7 days from study entry) were screened via the electronic medical record and recruited from surgical clinics, by a trained research assistant. | Scheduled reduced smoking (SRS) delivered by a handheld computer "QuitPal" + best practice (BP). The SRS schedule was tailored to current smoking habits with a quit date planned at 24 hours prior to inpatient admission, and the inter cigarette intervals are gradually increased over time to reduce daily smoking rate. As the smoking intervals increase, smokers practice behavioural coping with urges. The control group received BP only, which was a comprehensive multicomponent cessation treatment including tailored specialist telephone and bedside cessation counselling, self-help materials, and free NRT. There were 5 counselling sessions (2 pre-op, 1 during hospitalisation and 2 post-discharge). | Primary: 7-day point prevalence abstinence at 6 months verified by salivary cotinine. Secondary: abstinence at hospital admission (24-hr point prevalence) as verified by CO test, and 7-day point prevalence verified by salivary cotinine at 3 months postoperatively. |
| Ratner 2004 [^14^](#_ENREF_14)  RCT  (Canada) | Elective surgery (general; obstetrical/gynaecologic; cardiovascular; orthopaedic; urologic; vascular and other).  n=237 | Patients admitted for pre-surgical assessment (1-3 weeks before surgery) were screened for eligibility by registered nurses. | Registered nurse trained in smoking cessation provided AHCPR-based intervention in the PAC, hospital and post-surgery; including face-to-face counselling, printed materials and self-help cards, NRT, stress reduction aids and distractors, hotline number for further advice from the study. Telephone counselling post-surgery was provided weekly for the first month, and bi-weekly for the second and third months. The ninth and final call was made 16 weeks following discharge. The control group received standard hospital care. | Smoking abstinence at 12 months. |
| Shi 2013 [^15^](#_ENREF_15)  RCT  (USA) | Inpatient and outpatient elective surgery.  n=169 | As part of routine preoperative evaluation (POE) patients provide information on smoking behaviour. The median time from study assessment at POE to surgery was 1 day (IQR 1 to 3). Smokers were identified by clinical POE personnel on a convenience basis. Consent by study personnel was obtained after study procedures were completed. | In POE clinicians are encouraged to provide a message to all smokers to maintain preoperative abstinence. After completion of the evaluation, all smokers received brief advice (BA) to reinforce this message. Participants in the CO + BA group were informed (by a study coordinator) that their smoking status would be checked by exhaled CO monitoring on the day of surgery. Participants in the BA only group were instead provided a general stop smoking message (not specifically related to preoperative abstinence) to ensure the total time spent with study personnel was the same (5 minutes). | CO levels on the day of surgery. |
| Sorensen 2007 [^16^](#_ENREF_16)  RCT  (Denmark) | Hernia (open incisional or inguinal day-case herniotomy).  n=180 | Smokers scheduled for elective open incisional or inguinal day-case herniotomy were included (about 3 months before surgery). Recruiting method not described, although the involvement of a study nurse was mentioned. | Smokers were advised (by a study nurse) to stop smoking peri-operatively at least 1 month before the surgery and until removal of sutures 10 days after. Then they were randomised to: (1) standard group not contacted until the day of surgery; (2) telephone group who received a 10 minute call as a reminder 1 month before surgery; and (3) outpatient group with a 20 minute meeting in the outpatient clinic 1 month before surgery. | Smoking behaviour and post-operative wound infection 3 months after surgery. |
| Sorensen 2003 [^17^](#_ENREF_17)  RCT  (Denmark) | Open colonic or rectal surgery.  n=60 | Daily smokers were included (>2-3 weeks before surgery). The study was originally planned as a 5-centre trial of 300 smokers. After initiation 4 centres failed to enrol participants, and recruitment was continued at the remaining centre until an equal number of participants in both groups were enrolled (n=60). Unclear about how smokers were identified and recruited. | A project nurse advised smokers to abstain from smoking the following day until removal of sutures after surgery. Participants were also offered individual support and NRT until 24 h before surgery, including a telephone call the day after expected cessation and an additional visit in the outpatient clinic or in the participant's home. All participants were free to call for additional support during working hours. The control group were asked to maintain daily smoking habits preoperatively and during their stay in hospital. | Post-operative complications, 1 month post-operation. |
| Thomsen 2010 [^18^](#_ENREF_18)^,^ [^19^](#_ENREF_19)  RCT  (Denmark) | Breast cancer.  n=130 | Women scheduled for breast surgery were included (>3-7 days before surgery). Methods for identifying and recruiting smokers were not reported. | Trained counsellors provided a single motivational counselling session (45-90 minutes) 3-7 days before surgery aimed at supporting cessation from 2 days before to 10 days after surgery. Individualised NRT was offered free of charge for the recommended perioperative smoking cessation period. The control group received routine preoperative information. | Post-operative complications and smoking cessation at 12 months. |
| Warner 2011 [^20^](#_ENREF_20)  RCT  (USA) | Elective surgery (in-patient and out-patient).  n=300 | Current smokers were recruited from the POE in preparation for surgery on a convenience basis. Recruitment occurred when appropriate research and clinical personnel were available. Time from preoperative evaluation to surgery: median 1 day, IQR 1-4 days. | The 5 minute intervention included advice to quit, a description and brochure about the quitline services. If the quitline was contacted, participants were offered the first 45 minute counselling and up to 8 subsequent proactive sessions, up to 8 weeks of free NRT. The control group received advice to quit, the benefits of quitting to surgical outcomes, techniques to aid quitting including the quitline (although services were not described). | Use of a quitline service. Self-reported abstinence from cigarettes at 30 and 90 days post-surgery. |
| Warner 2005 [^21^](#_ENREF_21)  RCT  (USA) | Elective surgery.  n=121 | Smokers were recruited from patients evaluated at the POE in preparation for surgery. Methods for identifying and recruiting smokers were not reported. | NRT or placebo patches. Study personnel applied the first patches on the morning of surgery and up to 30 days after discharge. | Changes in psychological stress. Secondary: changes to post-operative smoking behaviour (6 months post-operatively). |
| Warner 2012 [^22^](#_ENREF_22)  Pilot RCT  (USA) | Elective surgery (in-patient and out-patient).  n=46 | Current smokers were recruited from the POE in preparation for surgery on a convenience basis. Recruiting method not reported. | Active nicotine or placebo lozenges. All smokers regardless of group received a brief (2 mins) behavioural intervention advising abstinence from smoking after 7 pm the night before surgery including the potential benefits of abstinence and asking them to consider using a lozenge at times when they would usually smoke. | Exhaled CO concentration immediately prior to surgery. |
| Wolfenden 2005 [^23-25^](#_ENREF_23)  RCT  (Australia) | Non-cardiac elective surgery.  n=210 | Patients at high risk were booked to attend a non-cardiac preoperative clinic 1-2 weeks before their scheduled procedure. Participants completed a computerised assessment and those identifying themselves as smokers were recruited by a research assistant. | A multi-component intervention including computerised tailored counselling, self-help material, brief cessation advice by nursing and anaesthetic staff, preoperative telephone counselling delivered by a trained research assistant. Dependent smokers (> 10 cigarettes/day) received free NRT preoperatively and during ward stay. The control group received usual care (clinic staff had the opportunity to provide quitting advice and prescribe NRT at their discretion). | Smoking abstinence (>24 h before admission, and 3-month point prevalence abstinence) and change in smoking habits. |
| Wong 2012 [^26^](#_ENREF_26)  RCT  (Canada) | Elective non-cardiac surgery (inpatient or outpatient).  n=286 | Smoking patients scheduled for surgery (8-30 days before the scheduled surgery), who met the eligibility criteria were recruited. Recruiting method not reported. | Varenicline or placebo for 12 weeks, started 1 week before the target quit date (i.e., 1 day before surgery). Both groups received in-hospital and telephone counselling (by trained research coordinators): the first in-person counselling session occurred in the preoperative clinic, and the second in-person session occurred before discharge or 24 hours after surgery. Telephone counselling was weekly for the first 4 weeks and at the end of 8 weeks. From 3-12 months, phone calls were every 4 weeks. | Primary outcome: 7-day point prevalence abstinence rate 12 months after surgery. |
| **Other non-RCT designs** | | | | |
| Backer 2007 [^27^](#_ENREF_27)  Not controlled, audit study  (Denmark) | Acute orthopaedic problems, principally fractures.  n=121 | On the day of admission, patients admitted to acute orthopaedic wards on weekdays were routinely asked about smoking habits as part of their medical history. A specially trained nurse in the morning briefing reviewed medical files to ensure smokers could be contacted. | A motivational counselling session by a trained nurse on the day of admission, followed by a 6 week smoking intervention programme at the Tobacco Cessation Clinic. | Acceptance of motivational counselling; quitting or reduction in smoking status; requesting admission to the Tobacco Cessation Clinic. |
| Browning 2000 [^28^](#_ENREF_28)  Controlled study  (USA) | Lung cancer surgery.  n=25 | Researchers recruited potential participants from a lung cancer surgery clinic's new patient schedule, during the first clinical consultation. | A nurse-delivered (with a personalized message from the surgeon) the AHCPR-based intervention, including leaflets, face-to-face and phone contact beginning at the first preoperative lung cancer surgery clinic. Behavioural and cognitive strategies were employed and NRT and Zyban were encouraged. Usual care participants received usual physician care including a strong message to quit smoking. | Self-reported and biochemical validated abstinence at 6 months post-surgery. |
| Haddock 1997 [^29^](#_ENREF_29)  Controlled, pilot, quasi-experimental  (UK) | General day surgery and gynaecology surgery.  n=60 | One research nurse implemented smoking cessation programme in surgical pre-admission clinics (7-14 days before surgery). Smokers who were willing to participate were eligible for the study. | A staff nurse knowledgeable about smoking behaviour and cessation provided the face-to-face intervention (20-40 minutes), including a variety of leaflets, advice on cessation aids with referral to community pharmacist or GP, a diary to help patients gain insights into their smoking behaviour, active listening, general and preoperative cessation advice, and self-assessment questionnaires related to smoking cessation when attending the clinic. The control group received usual care. | Changes in smoking behaviour; adoption of the programme; assessment of knowledge; levels of satisfaction. |
| Haile 2002 [^30^](#_ENREF_30)  Not controlled, pilot study  (Australia) | Non-cardiac surgery.  n=56 | Prior to booking in for surgery all patients were given a surgical risk assessment by their GP, surgeon or preadmission staff. Patients at high risk were required to attend the pre-admission clinic 2 weeks prior to surgery, and a research assistant determined eligibility. | A structured interactive computerized smoking cessation programme was provided to all smokers. It was tailored to the degree of nicotine dependence and stage of change; including: firm advice to quit; information on the surgical risks of smoking; setting a quit date; advice on smoking behaviour and adopting alternate behaviours and social support; and a recommendation to use NRT. | Self-reported smoking status at 9 month follow-up, feasibility and acceptability of computer delivery of smoking cessation advice. |
| Kozower 2010 [^31^](#_ENREF_31)  Not controlled, pilot study  (USA) | Thoracic surgery.  n=23 (preoperative) | Recruiting method not reported. Included preoperative (23), postoperative (11) and follow-up smokers (6). A clinical research coordinator was present to facilitate the study. | A brief office intervention (<10 min) from thoracic surgeon and a clinical research nurse: including discussion of smoking history, previous quit attempts, current motivation for quitting. Participants were offered tobacco co-dependence treatment and use of a free telephone quitline and written materials for their medication and quitline use. | Primary outcome: CO-validated 7-day abstinence at 3 months. |
| Kunzel 2012 [^32^](#_ENREF_32)  Not controlled, pilot study  (USA) | Urologic.  n=38 | Preoperatively (the mean interval to day of surgery was 25 days, median 11, range 1-131 days). Recruiting method not reported, although urologists and research staff performed the intervention. | Delivered by urologists, based on the AAR (Ask, Advise and Refer) strategy. Patients received strong advice to quit before surgery and in the long-term. They were given educational materials highlighting the benefits of cessation and were referred to a free smoking quit telephone line. | Self-reported abstinence, expired CO and urinary cotinine levels on the morning of surgery and at the first post-operative visit. |
| Moore 2005 [^33^](#_ENREF_33)  Not controlled, prospective cohort  (USA) | Urogynecologic.  n=233 | On their initial history and physical exam, patients who admitted to smoking were recruited (>1 month prior to surgery). Unclear about how smokers were identified. | Smokers were required to participate in a smoking cessation programme of their choice. Smoking cessation was for a minimum of 1 month before surgery and 1 month after surgery. No specific programme was recommended but participants were counselled regarding options: NRT, counselling; and/or medical therapy. | Post-operative complications. |
| Munday 1993 [^34^](#_ENREF_34)  Controlled study  (UK) | Elective surgery.  n=233 | At the time of outpatient consultation (>6 weeks prior to surgery), smokers were identified and the hospital notes were marked to enable the smokers to be identified when admitted to hospital. Control smokers were participants admitted from the waiting list for elective surgery who smoked but had not been given specific advice to stop. Unclear about who identified and recruited smokers. | A leaflet entitled "Smoking and Your Operation" produced in conjunction with the Health Education Authority. The leaflet described adverse effects of smoking and the reasons for stopping smoking before an operation. The leaflet advised participants to give up smoking at least 6 weeks before their operation. | Duration of abstinence before the operation. |
| Sachs 2012 [^35^](#_ENREF_35)  Controlled study  (Canada) | Elective surgery (excluding cardiovascular and neurosurgery and plastic.  n=714 | At pre-admission clinic (PAC), registration clerks identified patients who were current smokers and informed them about the programme. Eligible participants were asked by research staff if they would be willing to participate in the evaluation study. | The intensive Intervention consisted of brief Intervention (BI by trained researchers) plus motivational interviews (by a Batchelor-level psychologist) and NRT patches during admission. One motivational interview was offered during hospital stay (24-48 hr after surgery) and one by telephone two weeks after discharge. BI only was provided in the control group, consisted of a clear statement of the benefits of not smoking, a self-help manual, and a quit number helpline. | Self-reported current smoking status and quit attempts 6 months after surgery. |
| Shah 1984 [^36^](#_ENREF_36)  Controlled study  (UK) | Elective surgery.  n=200 | In the intervention group a letter was sent with the admission note to all patients; non-smokers were asked to disregard the letter. In the control group recruitment occurred on the day of the operation after recovery. Unclear about how smokers were identified and recruited. | Letter sent to all patients (smokers and non-smokers) strongly advising patients to stop smoking for 5 days before the operation. The control group received only an admission letter in the usual way. | Mean cigarette consumption and abstinence on the day of surgery. |
| Tonnesen 2010 [^37^](#_ENREF_37)  Before-after  (Denmark) | Any elective surgery.  n=57 | GP (199) were invited to identify daily smoking when referring them to surgery and to refer high risk patients to a preoperative risk reduction programme. However, only 2 patients were referred a few months after starting the programme, and additional efforts slightly increased referral to the programme (7/72). High risk patients for elective surgery were also identified at the department of orthopaedic surgery and surgical gastroenterology (method not reported). | Healthy lifestyle maintenance programme until the date of surgery which took place in the hospital out-patient clinics or in units in close relation to the surgical pathway; delivered by experts as co-ordinated by the Hospital Tobacco Cessation Clinic. | Number of high risk patients for elective surgery referred to a risk reduction programme by GPs. |
| Walker 2009 [^38^](#_ENREF_38)  Not controlled  (UK) | Forefoot surgery.  n=25 | Senior author (based at Orthopaedic Department) reviewed all patients prior to planed surgery (approximately 6 months prior to planned surgery). | About 6 months before the planned surgery, patients were given an outline of the risks of smoking pertaining to forefoot surgery. Smokers were advised to stop and recommended to see their GP for specific help and practical interventions. No further advice was given but was left to the patients and their GP. At the preoperative clinic this advice was reiterated. | Smoking status on admission, in post-operative clinics (1-2 weeks post surgery) and via telephone surgery at a mean of 12 months post-operatively. |
| Webb et al 2014 [^39^](#_ENREF_39)  Before-after  (Australia) | Non-obstetric elective surgery  n=347 | All adult patients (including smokers) at time of waiting list placement for non-obstetric (general, vascular, orthopaedics, gynaecology, ENT, and urology) elective surgery. | Quit-packs consisted of colour brochure, Quitline referral form and reply-paid envelop. It was sent to all wait list patients (non-smokers too) as this proved more cost-effective than identifying smokers and packaging their waiting list information separately. | Primary end point: abstinence for ≥1 month at surgery. |
| Wheatley 1977 [^40^](#_ENREF_40)  Controlled  (Australia) | Inguinal hernia repair.  n=15 | Patients (15 smokers and 15 non-smokers) were assigned arbitrarily to one of three groups. Recruiting methods not reported. | Preoperative respiratory care: (1) admission to hospital 2 days before surgery with routine preoperative ward breathing instruction; (2) admission to hospital 2 days before surgery and no smoking 5 days before surgery; (3) admission to hospital 5 days preoperatively and no smoking and intensive physiotherapy. | Post-operative complications. |

Note to Additional file 2:

**References of the included studies:**

1. Andrews K, Bale P, Chu J, Cramer A, Aveyard P. A randomized controlled trial to assess the effectiveness of a letter from a consultant surgeon in causing smokers to stop smoking pre-operatively. *Public health* 2006; **120**(4): 356-8.

2. Glasgow RE, Estabrooks PA, Marcus AC, et al. Evaluating initial reach and robustness of a practical randomized trial of smoking reduction. *Health Psychology* 2008; **27**(6): 780-8.

3. Glasgow RE, Gaglio B, Estabrooks PA, et al. Long-term results of a smoking reduction program. *Med Care* 2009; **47**(1): 115-20.

4. Lee SM, Landry J, Jones PM, Buhrmann O, Morley-Forster P. The effectiveness of a perioperative smoking cessation program: a randomized clinical trial. *Anesthesia and analgesia* 2013; **117**(3): 605-13.

5. Lindstrom D, Sadr Azodi O, Wladis A, et al. Effects of a perioperative smoking cessation intervention on postoperative complications: a randomized trial. *Annals of surgery* 2008; **248**(5): 739-45.

6. Lindström D, Wladis A, Pekkari K. The thioredoxin and glutaredoxin systems in smoking cessation and the possible relation to postoperative wound complications. *Wounds: A Compendium of Clinical Research & Practice* 2010; **22**(4): 88-93.

7. Sadr Azodi O, Lindstrom D, Adami J, et al. The efficacy of a smoking cessation programme in patients undergoing elective surgery: a randomised clinical trial. *Anaesthesia* 2009; **64**(3): 259-65.

8. McHugh F, Lindsay GM, Hanlon P, et al. Nurse led shared care for patients on the waiting list for coronary artery bypass surgery: a randomised controlled trial. *Heart (British Cardiac Society)* 2001; **86**(3): 317-23.

9. Moller AM, Villebro N, Pedersen T, Tonnesen H. Effect of preoperative smoking intervention on postoperative complications: a randomised clinical trial. *Lancet* 2002; **359**(9301): 114-7.

10. Villebro NM, Pedersen T, Moller AM, Tonnesen H. Long-term effects of a preoperative smoking cessation programme. *The clinical respiratory journal* 2008; **2**(3): 175-82.

11. Moller AM, Pedersen T, Villebro N, Norgaard P. Impact of lifestyle on perioperative smoking cessation and postoperative complication rate. *Prev Med* 2003; **36**(6): 704-9.

12. Myles PS, Leslie K, Angliss M, Mezzavia P, Lee L. Effectiveness of bupropion as an aid to stopping smoking before elective surgery: a randomised controlled trial. *Anaesthesia* 2004; **59**(11): 1053-8.

13. Ostroff JS, Burkhalter JE, Cinciripini PM, et al. Randomized Trial of a Presurgical Scheduled Reduced Smoking Intervention for Patients Newly Diagnosed With Cancer. *Health psychology : official journal of the Division of Health Psychology, American Psychological Association* 2013.

14. Ratner PA, Johnson JL, Richardson CG, et al. Efficacy of a smoking-cessation intervention for elective-surgical patients. *Res Nurs Health* 2004; **27**(3): 148-61.

15. Shi Y, Ehlers S, Hinds R, Baumgartner A, Warner DO. Monitoring of exhaled carbon monoxide to promote preoperative smoking abstinence. *Health psychology : official journal of the Division of Health Psychology, American Psychological Association* 2013; **32**(6): 714-7.

16. Sorensen LT, Hemmingsen U, Jorgensen T. Strategies of smoking cessation intervention before hernia surgery--effect on perioperative smoking behavior. *Hernia : the journal of hernias and abdominal wall surgery* 2007; **11**(4): 327-33.

17. Sorensen LT, Jorgensen T. Short-term pre-operative smoking cessation intervention does not affect postoperative complications in colorectal surgery: a randomized clinical trial. *Colorectal disease : the official journal of the Association of Coloproctology of Great Britain and Ireland* 2003; **5**(4): 347-52.

18. Thomsen T, Tonnesen H, Okholm M, et al. Brief smoking cessation intervention in relation to breast cancer surgery: a randomized controlled trial. *Nicotine Tob Res* 2010; **12**(11): 1118-24.

19. Thomsen T, Esbensen BA, Samuelsen S, Tonnesen H, Moller AM. Brief preoperative smoking cessation counselling in relation to breast cancer surgery: a qualitative study. *European journal of oncology nursing : the official journal of European Oncology Nursing Society* 2009; **13**(5): 344-9.

20. Warner DO, Klesges RC, Dale LC, et al. Clinician-delivered intervention to facilitate tobacco quitline use by surgical patients. *Anesthesiology* 2011; **114**(4): 847-55.

21. Warner DO, Patten CA, Ames SC, Offord KP, Schroeder DR. Effect of nicotine replacement therapy on stress and smoking behavior in surgical patients. *Anesthesiology* 2005; **102**(6): 1138-46.

22. Warner DO, Kadimpati S. Nicotine lozenges to promote brief preoperative abstinene from smoking: pilot study. *Clin Health Promot* 2012; **2**(3): 85-8.

23. Wolfenden L, Wiggers J, Knight J, et al. A programme for reducing smoking in pre-operative surgical patients: randomised controlled trial. *Anaesthesia* 2005; **60**(2): 172-9.

24. Wolfenden L, Wiggers J, Knight J, et al. Increasing smoking cessation care in a preoperative clinic: a randomized controlled trial. *Prev Med* 2005; **41**(1): 284-90.

25. Wolfenden L, Wiggers J, Campbell E, Knight J. Pilot of a preoperative smoking cessation intervention incorporating post-discharge support from a Quitline. *Health promotion journal of Australia : official journal of Australian Association of Health Promotion Professionals* 2008; **19**(2): 158-60.

26. Wong J, Abrishami A, Yang Y, et al. A perioperative smoking cessation intervention with varenicline: a double-blind, randomized, placebo-controlled trial. *Anesthesiology* 2012; **117**(4): 755-64.

27. Backer V, Nelbom BM, Duus BR, Tonnesen H. Introduction of new guidelines for emergency patients: motivational counselling among smokers. *The clinical respiratory journal* 2007; **1**(1): 37-41.

28. Browning KK, Ahijevych KL, Ross P, Jr., Wewers ME. Implementing the Agency for Health Care Policy and Research's Smoking Cessation Guideline in a lung cancer surgery clinic. *Oncol Nurs Forum* 2000; **27**(8): 1248-54.

29. Haddock J, Burrows C. The role of the nurse in health promotion: an evaluation of a smoking cessation programme in surgical pre-admission clinics. *Journal of advanced nursing* 1997; **26**(6): 1098-110.

30. Haile MJ, Wiggers JH, A DS, Knight J, Considine RJ, Moore K. Novel strategy to stop cigarette smoking by surgical patients: pilot study in a preadmission clinic. *ANZ journal of surgery* 2002; **72**(9): 618-22.

31. Kozower BD, Lau CL, Phillips JV, Burks SG, Jones DR, Stukenborg GJ. A thoracic surgeon-directed tobacco cessation intervention. *The Annals of thoracic surgery* 2010; **89**(3): 926-30+ADs- discussion 30.

32. Kunzel B, Cabalza J, Faurot M, et al. Prospective pilot study of smoking cessation in patients undergoing urologic surgery. *Urology* 2012; **80**(1): 104-9.

33. Moore S, Mills BB, Moore RD, Miklos JR, Mattox TF. Perisurgical smoking cessation and reduction of postoperative complications. *American journal of obstetrics and gynecology* 2005; **192**(5): 1718-21.

34. Munday IT, Desai PM, Marshall CA, Jones RM, Phillips ML, Rosen M. The effectiveness of pre-operative advice to stop smoking: a prospective controlled trial. *Anaesthesia* 1993; **48**(9): 816-8.

35. Sachs R, Wild TC, Thomas L, Hammal F, Finegan BA. Smoking cessation interventions in the pre-admission clinic: Assessing two approaches. *Canadian Journal of Anesthesia* 2012; **59**(7): 662-9.

36. Shah MV, Watkins G, Latto IP. The effect of written advice on preoperative cigarette consumption. *Ann R Coll Surg Engl* 1984; **66**(6): 436-7.

37. Tonnesen H, Faurschou P, Ralov H, Molgaard-Nielsen D, Thomas G, Backer V. Risk reduction before surgery. The role of the primary care provider in preoperative smoking and alcohol cessation. *BMC health services research* 2010; **10**: 121.

38. Walker NM, Morris SA, Cannon LB. The effect of pre-operative counselling on smoking patterns in patients undergoing forefoot surgery. *J Foot Ankle Surg* 2009; **15**(2): 86-9.

39. Webb AR, Robertson N, Sparrow M, Borland R, Leong S. Printed quit-pack sent to surgical patients at time of waiting list placement improved perioperative quitting. *ANZ journal of surgery* 2014; **84**(9): 660-4.

40. Wheatley IC, Hardy KJ, Barter CE. An evaluation of preoperative methods of preventing postoperative pulmonary complications. *Anaesth Intensive Care* 1977; **5**(1): 56-9.
