# Supplementary material for: Identifying and recruiting smokers for preoperative smoking cessation—a systematic review of methods reported in published studies
Source: Syst Rev. 2015 Nov 11;4:157. doi: 10.1186/s13643-015-0152-x (PMC4642619; doi:10.1186/s13643-015-0152-x)
Supplement: Additional file 3: — List of excluded studies after full text examination.(DOCX 21.5 kb) [file 13643_2015_152_MOESM3_ESM.docx]

**List of excluded studies after full text examination**

| # | **Study** | **Reason for Exclusion** |
| --- | --- | --- |
| 1 | Abdelaziz, M., et al., A multidisciplinary complex perioperative intervention to reduce complications and enhance recovery after lung resection surgery. Lung Cancer, 2011. 71: p. S26. | Abstract only |
| 2 | Abrishami, A., Prevalence of smoking and stage of readiness for smoking cessation in surgical patients. Canadian Journal of Anesthesia, 2010. 57: p. S44-S45. | Abstract only |
| 3 | Adams, K., et al., Patients on ERAS following colorectal resection have a reduced length of stay, without increased readmissions, re-operations or complications rates. Colorectal Disease, 2011. 13: p. 11. | Abstract only |
| 4 | Finegan, B., Clearing the smoke: Institutionalizing a perioperative smoking cessation program. Canadian Journal of Anesthesia, 2010. 57: p. S35. | Abstract only |
| 5 | Kerr, A., et al., Rehabilitation for operated lung cancer programme: 18-month outcomes. Interactive Cardiovascular and Thoracic Surgery, 2013. 17: p. S120. | Abstract only |
| 6 | Kunzel, B., et al., Prospective study of smoking cessation in patients undergoing urologic surgery. Journal of Urology, 2011. 1): p. e23. | Abstract only |
| 7 | McDonnell, K.K., A decision aid to improve smoking abstinence and hrql for families facing cancer, 2014, ProQuest Information & Learning: US. | Abstract only |
| 8 | Moller, A., et al., Pre-operative nicotine replacement and smoking cessation counselling reduce post-operative complications. Evidence-Based Healthcare, 2002. 6(4): p. 190-191. | Abstract only |
| 9 | Moller, A.M., The impact of smoking on the peri-operative course and the effect of pre-operative smoking intervention: PhD abstract. Acta Anaesthesiologica Scandinavica, 2007. 51(3): p. 380-381. | Abstract only |
| 10 | Ostroff, J., et al., Randomized trial of a pre-surgical, scheduled reduced smoking intervention for patients newly diagnosed with cancer. Psycho-Oncology, 2013. 22: p. 74-75. | Abstract only |
| 11 | Paci, E., The ITALUNG study and the state of art of randomized screening trials in Europe. Cancer Prevention Research, 2011. 1). | Abstract only |
| 12 | Paci, E., et al., The italung randomised trial: Results of the screening rounds and perspectives. Journal of Thoracic Oncology, 2011. 2): p. S131-S132. | Abstract only |
| 13 | Pomeshkina, S., I.V. Borovik, and O.L. Barbarash, Adherence to non-medication treatment in patients undergoing coronary artery bypass surgery. European Heart Journal, 2013. 34: p. 306. | Abstract only |
| 14 | Steliga, M.A., et al., Implementation of a tobacco treatment program within a multidisciplinary thoracic oncology surgery clinic. Journal of Thoracic Oncology, 2013. 8: p. S1295-S1296. | Abstract only |
| 15 | Araco, A., et al., Wound infections in aesthetic abdominoplasties: The role of smoking. Plastic and Reconstructive Surgery, 2008. 121(5): p. 305e-310e. | Not preoperative smoking cessation intervention |
| 16 | Assadian, A., et al., Prevalence of patients continuing to smoke after vascular interventions. Wiener Klinische Wochenschrift, 2006. 118(7-8): p. 212-6. | Not preoperative smoking cessation intervention |
| 17 | Balduyck, B., et al., The effect of smoking cessation on quality of life after lung cancer surgery. European Journal of Cardio-Thoracic Surgery, 2011. 40(6): p. 1432-7; discussion 1437-8. | Not preoperative smoking cessation intervention |
| 18 | Barrera, R., et al., Smoking and timing of cessation: impact on pulmonary complications after thoracotomy. Chest, 2005. 127(6): p. 1977-83. | Not preoperative smoking cessation intervention |
| 19 | Bluman, L.G., et al., Preoperative smoking habits and postoperative pulmonary complications. Chest, 1998. 113(4): p. 883-9. | Not preoperative smoking cessation intervention |
| 20 | Bobbio, A., et al., Preoperative pulmonary rehabilitation in patients undergoing lung resection for non-small cell lung cancer. European Journal of Cardio-thoracic Surgery, 2008. 33(1): p. 95-98. | Not preoperative smoking cessation intervention |
| 21 | Bolukbas, S., et al., Short-term effects of inhalative tiotropium/formoterol/budenoside versus tiotropium/formoterol in patients with newly diagnosed chronic obstructive pulmonary disease requiring surgery for lung cancer: a prospective randomized trial. European Journal of Cardio-Thoracic Surgery, 2011. 39(6): p. 995-1000. | Not preoperative smoking cessation intervention |
| 22 | Chahine, R., A. Abchee, and P. Zalloua, Nicotine metabolism in healthy smokers and patients with cardiovascular diseases. Molecular & Cellular Biochemistry, 2005. 280(1-2): p. 241-4. | Not preoperative smoking cessation intervention |
| 23 | Geddes, D., et al., Effect of lung-volume-reduction surgery in patients with severe emphysema. New England Journal of Medicine, 2000. 343(4): p. 239-45. | Not preoperative smoking cessation intervention |
| 24 | Gravante, G., et al., Wound infections in post-bariatric patients undergoing body contouring abdominoplasty: The role of smoking. Obesity Surgery, 2007. 17(10): p. 1325-1331. | Not preoperative smoking cessation intervention |
| 25 | Mason, D.P., et al., Impact of smoking cessation before resection of lung cancer: a Society of Thoracic Surgeons General Thoracic Surgery Database study. Annals of Thoracic Surgery, 2009. 88(2): p. 362-70; discussion 370-1. | Not preoperative smoking cessation intervention |
| 26 | Nasell, H., et al., Effect of smoking cessation intervention on results of acute fracture surgery: a randomized controlled trial. Journal of Bone & Joint Surgery - American Volume, 2010. 92(6): p. 1335-42. | Not preoperative smoking cessation intervention |
| 27 | Quist-Paulsen, P., P.S. Bakke, and F. Gallefoss, Predictors of smoking cessation in patients admitted for acute coronary heart disease. European Journal of Cardiovascular Prevention & Rehabilitation, 2005. 12(5): p. 472-7. | Not preoperative smoking cessation intervention |
| 28 | Quist-Paulsen, P. and F. Gallefoss, Randomised controlled trial of smoking cessation intervention after admission for coronary heart disease. BMJ, 2003. 327(7426): p. 1254-7. | Not preoperative smoking cessation intervention |
| 29 | Rice, V.H., et al., A comparison of nursing interventions for smoking cessation in adults with cardiovascular health problems. Heart & Lung, 1994. 23(6): p. 473-86. | Not preoperative smoking cessation intervention |
| 30 | Storm-Versloot, M.N., et al., The number of smokers needed to screen and treat in a smoking cessation programme. European Journal of Cardiovascular Prevention & Rehabilitation, 2009. 16(6): p. 669-76. | Not preoperative smoking cessation intervention |
| 31 | Warner, M.A., et al., Role of preoperative cessation of smoking and other factors in postoperative pulmonary complications: a blinded prospective study of coronary artery bypass patients. Mayo Clinic Proceedings, 1989. 64(6): p. 609-16. | Not preoperative smoking cessation intervention |
| 32 | Zalesskiy, V.N., I.A. Belousova, and G.V. Frolov, Laser-acupuncture reduces cigarette smoking: A preliminary report. Acupuncture and Electro-Therapeutics Research, 1983. 8(3-4): p. 297-302. | Not preoperative smoking cessation intervention |
| 33 | Puura, A., Transdermal nicotine increases heart rate after endotracheal intubation. Methods and Findings in Experimental and Clinical Pharmacology, 2003. 25(5): p. 383-385. | Not preoperative smoking cessation intervention |
| 34 | Park, E.R., et al., A smoking cessation intervention for thoracic surgery and oncology clinics: a pilot trial.[Erratum appears in J Thorac Oncol. 2011 Aug;6(8):1454]. Journal of Thoracic Oncology: Official Publication of the International Association for the Study of Lung Cancer, 2011. 6(6): p. 1059-65. | Not preoperative smoking cessation intervention |
| 35 | Rissel, C., A. Salmon, and A.M. Hughes, Evaluation of a (pilot) stage-tailored brief smoking cessation intervention among hospital patients presenting to a hospital pre-admission clinic. Australian health review : a publication of the Australian Hospital Association, 2000. 23(3): p. 83-93. | Not preoperative smoking cessation intervention |
| 36 | Chan, L.K., S. Withey, and P.E. Butler, Smoking and wound healing problems in reduction mammaplasty: is the introduction of urine nicotine testing justified?.[Erratum appears in Ann Plast Surg. 2006 Apr;56(4):358]. Annals of Plastic Surgery, 2006. 56(2): p. 111-5. | Retrospective |
| 37 | Cooley, M.E., et al., Smoking cessation is challenging even for patients recovering from lung cancer surgery with curative intent. Lung Cancer, 2009. 66(2): p. 218-25. | Retrospective |
| 38 | Gravante, G., et al., Postoperative wound infections after breast reductions: The role of smoking and the amount of tissue removed. Aesthetic Plastic Surgery, 2008. 32(1): p. 25-31. | Retrospective |
| 39 | Ngaage, D.L., et al., The impact of the duration of mechanical ventilation on the respiratory outcome in smokers undergoing cardiac surgery. Cardiovascular Surgery, 2002. 10(4): p. 345-50. | Retrospective |
| 40 | Warner, M.A., M.B. Divertie, and J.H. Tinker, Preoperative cessation of smoking and pulmonary complications in coronary artery bypass patients. Anesthesiology, 1984. 60(4): p. 380-383. | Retrospective |
| 41 | Anonymous: Smoking cessation interventions and strategies. Australian Nursing Journal, 2008. 16(6): p. 29-32. | Review/comment |
| 42 | Allen, G., Evidence for practice. Smoking-cessation intervention for surgical patients. AORN Journal, 2005. 81(2): p. 425-426. | Review/comment |
| 43 | Aveyard, P. and B. Dautzenberg, Temporary abstinence from smoking prior to surgery reduces harm to smokers. International Journal of Clinical Practice, 2010. 64(3): p. 285-8. | Review/comment |
| 44 | Clair, C. and N.A. Rigotti, Stopping smoking in the weeks prior to surgery has no effect on the risk of postoperative complications. Evidence-Based Medicine, 2012. 17(3): p. 101-102. | Review/comment |
| 45 | Ehrlich, A., Evidence-based medicine. Preoperative smoking cessation reduces risk of postoperative complications. Clinical Advisor for Nurse Practitioners, 2009. 12(8): p. 67-67. | Review/comment |
| 46 | Fentiman, I.S., Smoking cessation before surgery: not worth a light? International Journal of Clinical Practice, 2010. 64(3): p. 289-91. | Review/comment |
| 47 | Ihsan, K.M., et al., Perioperative management of chronic respiratory disease. Journal of Perioperative Practice, 2012. 22(10): p. 324-327. | Review/comment |
| 48 | Barone, M., A. Cogliandro, and P. Persichetti, Plastic surgery and smoking: a prospective analysis of incidence, compliance, and complications. Plastic & Reconstructive Surgery, 2013. 132(4): p. 686e-687e. | Review/comment |
| 49 | Beyea, S.C., Evidence for practice. Preoperative smoking intervention decreases postoperative complications. AORN Journal, 2002. 76(3): p. 520-520. | Review/comment |
| 50 | Iida, H., et al., Preoperative smoking cessation and smoke-free policy in a university hospital in Japan. Canadian Journal of Anesthesia, 2008. 55(5): p. 316-318. | Review/comment |
| 51 | Julian, D.G., Smoking and coronary artery bypass surgery. British Heart Journal, 1994. 72(1): p. 9-11. | Review/comment |
| 52 | Khullar, D., S.A. Schroeder, and J. Maa, Helping smokers quit around the time of surgery. JAMA, 2013. 309(10): p. 993-4. | Review/comment |
| 53 | Kozak, E., A preoperative smoking intervention decreased postoperative complications in elective knee or hip replacement. ACP Journal Club, 2002. 137(1): p. 7-7. | Review/comment |
| 54 | Lee, M.J., Optimizing the safety of surgery, before surgery. Clinical Orthopaedics and Related Research, 2014. 472(3): p. 809-811. | Review/comment |
| 55 | Maa, J., D. Warner, and S. Schroeder, What surgeons can do to reduce the impact of smoking on surgical outcomes. Bulletin of the American College of Surgeons, 2009. 94(11): p. 21-5. | Review/comment |
| 56 | Murray, E.W., A preoperative smoking intervention decreased postoperative complications in elective knee or hip replacement. Evidence Based Nursing, 2002. 5(3): p. 84-84. | Review/comment |
| 57 | Peters, M.J., L.C. Morgan, and L. Gluch, Smoking cessation and elective surgery: the cleanest cut. Medical Journal of Australia, 2004. 180(7): p. 317-8. | Review/comment |
| 58 | Slatore, C.G., D.H. Au, and W. Hollingworth, Cost-effectiveness of a smoking cessation program implemented at the time of surgery for lung cancer. Journal of Thoracic Oncology: Official Publication of the International Association for the Study of Lung Cancer, 2009. 4(4): p. 499-504. | Review/comment |
| 59 | Vieira Cavichio, B., et al., Duration of smoking cessation for the prevention of surgical wound healing complications. Revista da Escola de Enfermagem da USP, 2014. 48(1): p. 170-176. | Review/comment |
| 60 | Wein, R.O., Preoperative smoking cessation: Impact on perioperative and long-term complications. Archives of Otolaryngology - Head and Neck Surgery, 2009. 135(6): p. 597-601. | Review/comment |
| 61 | Wolfenden, L., et al., Providing comprehensive smoking cessation care to surgical patients: the case for computers. Drug & Alcohol Review, 2009. 28(1): p. 60-5. | Review/comment |
