# Supplementary material for: Identifying and recruiting smokers for preoperative smoking cessation—a systematic review of methods reported in published studies
Source: Syst Rev. 2015 Nov 11;4:157. doi: 10.1186/s13643-015-0152-x (PMC4642619; doi:10.1186/s13643-015-0152-x)
Supplement: Additional file 4: — Study selection flow diagram. (DOCX 27.8 kb) [file 13643_2015_152_MOESM4_ESM.docx]

**Study selection flow diagram**
